# *Dendrobium:* Sources of Active Ingredients to Treat Age-Related Pathologies

**DOI:** 10.14336/AD.2017.0214

**Published:** 2017-12-01

**Authors:** Veronika Cakova, Frederic Bonte, Annelise Lobstein

**Affiliations:** ^1^Université de Strasbourg, CNRS, LIT UMR 7200, F-67000 Strasbourg, France; ^2^LVMH Recherche, F-45800 Saint Jean de Braye, France

**Keywords:** *Dendrobium*, aging, anticancer, immunomodulatory, neuroprotective, anti-diabetic

## Abstract

*Dendrobium* represents one of the most important orchid genera, ornamentally and medicinally.* Dendrobiums* are sympodial epiphytic plants, which is a name they are worthy of, the name coming from Greek origin: "dendros", tree, and "bios", life. *Dendrobium* species have been used for a thousand years as first-rate herbs in traditional Chinese medicine (TCM). They are source of tonic, astringent, analgesic, antipyretic, and anti-inflammatory substances, and have been traditionally used as medicinal herbs in the treatment of a variety of disorders, such as, nourishing the stomach, enhancing production of body fluids or nourishing Yin. The Chinese consider *Dendrobium* as one of the fifty fundamental herbs used to treat all kinds of ailments and use *Dendrobium* tonic for longevity. This review is focused on main research conducted during the last decade (2006-2016) on *Dendrobium* plants and their constituents, which have been subjected to investigations of their pharmacological effects involving anticancer, anti-diabetic, neuroprotective and immunomodulating activities, to report their undeniable potential for treating age-related pathologies.

Orchidaceae is the largest botanical family of flowering plants. Among about 27,800 orchid species, *Dendrobium* represents one of the most important genera, ornamentally and medicinally. With more than 1,500 species (www.theplantlist.org), *Dendrobium* is the second largest orchid genus after *Bulbophyllum,* widely distributed throughout Asia, Europe and Australia. *Dendrobiums* are sympodial epiphytic plants, which is a name they are worthy of, the name coming from Greek origin: "dendros", tree, and "bios", life. In Japan, since the Antiquity, *Dendrobium moniliforme* ("Fu-ran") has been known as "the orchid that gives long life to men". *Dendrobium* species have been used for a thousand years as first-rate herbs and a prized folk medicine in India and China [1]. In traditional Chinese medicine (TCM), they are source of tonic, astringent, analgesic, antipyretic, and anti-inflammatory substances, and have been traditionally used as medicinal herbs in the treatment of a variety of disorders, such as, nourishing the stomach, enhancing production of body fluids or nourishing Yin. A common name "Shihu" is used for thirty *Dendrobium* species (*D. nobile*, *D. chrysotoxum*, *D. fimbriatum* and other related *Dendrobium* species) or "Tiepi shihu" for *D. officinale*. About seventy *Dendrobium* species are found in China, but only two monographs are found in Chinese Pharmacopoeia (2010 edition) [2].

In light of their traditional importance as medicinal plants, knowledge on the constituents of various *Dendrobium* species and their pharmacological activities has been growing, and methodologies have been developed for the effective propagation of members of this genus or for the authentication of *Dendrobium* drugs from its adulterants [3, 4]. In fact, the Chinese consider *Dendrobium* as one of the fifty fundamental herbs used to treat all kinds of ailments and use *Dendrobium* tonic for longevity [2, 3]. Indeed, a large number of pharmacological activities were assigned to different *Dendrobium* species, such as anti-inflammatory [5], anti-platelet aggregation [6], hepatoprotective [7], anti-fibrotic [8], anti-viral [9], anti-fungal [10], antimicrobial [11], antioxidant [12-14], anti-diabetic [15], neuroprotective [16], immunomodulatory and anticancer [17]. The scientific literature reports various studies on extracts from *Dendrobium* plants, as well as on their isolates. The most active constituents are polysaccharides, phenanthrene derivatives and alkaloids [16, 18, 19].

Aging is a normal physiological process, which represents the accumulation of changes in human organisms. Thanks to scientific progress and awareness of factors related to life style, the population is becoming older. The other side of the coin is that aging is one of the most important risk factor for the break out of many human diseases and people die from age-related disorders [20]. Aging successfully became a research and public health priority for the 21^st^ century [21]. In this context, researchers worldwide have been focusing their investigations on mechanisms involved in aging, especially in its related pathologies. The ones, which are often explored, are cancer, neurodegenerative diseases and diabetes mellitus as they are widely associated to elderly. Moreover, aging can be examined from an immunological point of view, because it is generally accepted, that the immune system does not work as well as we grow older and becomes altered with age. Therefore, the disturbed and weakened immunity can be a cause of disease among elderly [22].

Knowing that *Dendrobium* drugs have been traditionally used in TCM preparations as a universal remedy, they are worth investigating by scientists who are looking for new natural ingredients, which could become a main feature in healthy aging. This review is focused on the main research conducted during the last decade (2006-2016) on *Dendrobium* plants and their constituents, which have been subjected to investigations of their pharmacological effects involving cancer-related, anti-diabetic, neuroprotective and immunomodulating activities, in search for new ingredients to treat age-related pathologies.

## Anticancer effects

In the search of new strategies for fighting cancer, *Dendrobium* species as well as their isolates, have been studied as a valuable natural source of promising anticancer agents. Due to the high diversity of types of cancer, specific mechanisms of action have been explored to assign to *Dendrobium* spp. cytotoxic, anti-proliferative, anti-metastatic, antitumor, anti-genotoxic, antimigratory or apoptotic properties *in vitro* or *in vivo* and suggest their potential anticancer effects. Recently, *D. candidum* methanolic extract (DCME) was investigated for these effects on colon cancer. *In vitro*, HCT-116 human colon cancer cells were treated with DCME and its cytotoxic effect was evaluated using a MTT assay [23]. At 0.25, 0.5 and 1.0 mg/mL, DCME showed a cancer cell survival rate of 69%, 41% and 16%, respectively, by induction of apoptosis. Even if some studies have showed preliminarily anticancer effects of *D. candidum*, the specific mechanisms have not been fully clarified. Indeed, the cell proliferation is the most important characteristic of the tumor cells and could be indicated by the cell viability. Thus, human breast cancer cell line (MCF-7) and normal breast epithelial cell line MCF10A were used to observe the cell viability and explore the anti-proliferative effects of *D. candidum in vitro* [17]. The inhibition of the MCF-7 cell viability by enhancing the cell cycle arrest in G2/M phase and regulating the key biomarkers (tumor growth-associated biomarkers, including Erα, 1GPBP2, IGFBP4, GATA3, and apoptosis-associated biomarkers, including ELF5, p53, p21, p18, CDH1, CDH2, and p12) in breast cancer cells was demonstrated. In addition, treatment with *D. candidum* at any concentration and any time point caused no inhibitory effect on cell proliferation of MCF10A cell line, suggesting its selectivity against MCF-7 breast cancer cell proliferation. *Dendrobium formosum* leaf ethanolic extract showed antitumor activity on T-cell (Dalton’s) lymphoma. *In vitro*, the extract induced cytotoxic response in a concentration-dependent manner, with an IC_50_ value at 350 µg/mL, against Dalton’s lymphoma (DL) cells [24]. Moretti et al. (2013) assessed anti-genotoxic effect of *D. speciosum* on the liver hepatocellular carcinoma cell line (HepG2) [25]. The stems extract did not induce DNA strand breakage in the range of concentration tested (2.5-100.0 µg/mL), whereas at the higher concentration tested (100 µg/mL) the leaves extract caused a significant increase in the extent of DNA damage, thus indicating a genotoxic effect. However, when testing the *D. speciosum* stems extract for anti-genotoxicity, low doses result in enhanced activity, whereas higher doses result in no effect. Anti-proliferative effects were assigned to alcalase-derived polypeptides of *Dendrobium catenatum* against human liver (HepG2), gastric (SGC-7901) and breast (MCF-7) cancer cell lines [26]. The best anti-proliferative activity *in vitro* with a percentage of inhibitions of 73.38%, 78.91% and 86.8% against HepG2, SGC-7901 and MCF-7 cancer cells, respectively, were obtained at 500 µg/mL. The three most abundant peptides (RHPFDGPLLPPGD, RCGVNAFLP KSYLVHFGWKLLFHFD and KPEEVGGAGDRWTC, determined by *de novo* sequencing) were chemically synthesized and their anti-proliferative activities *in vitro* were also confirmed.

Studies reporting anticancer effects of *Dendrobium* isolates focus mostly on bibenzyl and phenanthrene derivatives. 3,4,3’-trimethoxy-5,4’-dihydroxybibenzyl (DTB), aloifoll and 5,3’-dihydroxy-3,4-dimethoxy-bibenzyl, along with a lignin glycoside longicornuol A, from *D. sinense* showed different degrees of cytotoxicity on SGC-7901, human hepatoma (BEL-7402) and chronic myelogenous leukemia (K562) cell lines. The three bibenzyls were cytotoxic against SGC-7901 (IC_50_ from 7.8±0.05 to 16.7±0.4 µM), while longicornuol A was more cytotoxic against BEL-7402 (IC_50_ 10.0±0.4 µM) and K562 (10.3±0.1 µM) [27]. Chaotham et al. (2014) focused their studies on 4,5,4’-trihydroxy-3,3’dimethoxybibenzyl (TDB) from *Dendrobium ellipsophyllum* and its anti-metastatic effect on lung cancer cells (H292) [28]. TDB inhibited epithelial-to-mesenchymal transition and sensitization of lung cancer cells to anoikis. Apoptosis induced by cell detachment was increased in TDB-treated cells and the formation of tumor in anchorage-independent growth was found to be reduced in response to the compound. In a more recent study, it has been demonstrated that the reduction of migration and invasion of H292 cells by TDB was reduced by decreasing migration-regulating proteins, including integrins αv, α4, β1, β3 and β5, as well as activated focal adhesion kinase (pFAK), activated Ras-related C3 botulinum toxin substrate 1 (Rac1-GTP) and cell division control protein 42 (Cdc42) [29]. Fourteen bibenzyls and bibenzyl dimers from *D. fimbriatum* were tested for their cytotoxic activity against five human cancer cell lines: human promyelocytic leukemia cells (HL-60), hepatocellular carcinoma cells (SMMC-7721), human alveolar basal epithelial cells (A-549), breast cancer cell line (MCF-7) and colorectal adenocarcinoma cell line (SW480) [30]. Among these bibenzyl derivatives, fimbriadimerbibenzyl A, B, E, F and G exhibited broad-spectrum and moderate cytotoxicity with IC_50_ values range from 5.85 to 21.23 µM. Moscatilin and 4-(3-hydroxy-4-methoxyphenetyl)-2,6-dimethoxylphenol were the most cytotoxic compounds with IC_50_ values range from 2.2 to 12.3 µM against all the cell lines, suggesting their tumor inhibitory activity. Amongst other studies on anticancer agents from *Dendrobium* plants, a MeOH extract obtained from a whole plant of *D. brymerianum* was evaluated and found to possess significant cytotoxicity against human non-small lung cancer H460 cells, showing 80% inhibition at a concentration of 50 µg/mL [31]. The investigation of the plant led to the identification of its cytotoxic components and to the determination of their antimigratory activity by wound-healing assay. Moscatilin, gigantol, lusianthridin and dendroflorin exhibited cytotoxic properties (IC_50_ < 200 µg/mL) and all of them inhibited the migration of the cells across the wound space in a time-dependent manner. Gigantol was the weakest antimigratory agent despite its strong cytotoxicity against H460 cells. During the first 24hr, dendroflorin appeared to be the strongest antimigratory compound. However, after 48hr, moscatilin became the most potent compound, as a result of the substantial increase of its activity during the period of 24-48hr. Moscatilin, isolated for the first time from *D. moscatum*, was also found from *Dendrobium pulchellum*. Its ability to attenuate migration and invasion in human lung cancer H23 cells was associated with an attenuation of endogenous reactive oxygen species (ROS), in which hydroxyl radical (OH•) was identified as a dominant species [32]. Moscatilin from *D. pulchellum*, together with chrysotobibenzyl, chrysotoxine and crepidatin showed the ability to facilitate anoikis (a form of programmed cell death) and inhibit the growth of lung cancer cells in anchorage-independent condition, thus they disclosed the inhibitory effect on cancer cell metastasis [33]. The effects of moscatilin isolated from *D. loddigesii* were assessed on VEGF and bFGF-induced angiogenesis in cultured human umbilical vein endothelial cells (HUVECs) *in vitro* and *in vivo* [34]. It significantly inhibited growth of lung cancer cell line A549 and suppressed growth factor-induced neovascularization. Moreover, VEGF- and bFGF-induced cell proliferation, migration and tube formation of HUVECs was markedly inhibited by moscatilin. Furthermore, this compound was found to induce apoptosis of colorectal cancer HCT-116 cells via tubulin depolymerization and DNA damage stress which leads to the activation of c-Jun NH2-terminal protein kinase and mitochondria-involved intrinsic apoptosis pathway [35]. Moscatilin also inhibits growth and induces apoptosis and mitotic catastrophe in human esophageal cancer cells, including squamous cell carcinoma (SCC) and adenocarcinoma (ADC) derived cell lines [36]. Apoptotic, anti-migratory and cytotoxic properties were also assigned to gigantol, isolated from *D. draconis*, Charoenrungruang et al. (2014) demonstrated the inhibitory effect of gigantol on H292 and H460 cell movement, down-regulation of caveolin-1, activation of ATP-dependent tyrosinase kinase, and cell division cycle 42, thereby suppressing filopodia formation (in nontoxic doses of 0-20 µM) [37]. A more recent study demonstrates that gigantol mediates lung cancer cells apoptosis via mitochondrial-dependent mechanism [38]. Moreover, Unahabhokha et al. (2016) demonstrated for the first time that gigantol significantly decreases lung cancer cells viability in a detached condition through anoikis and anchorage-independent assays. Western blotting analysis revealed that this compound greatly decreases epithelial to mesenchymal transition (EMT) markers, including N-cadherin, vimentin, and Slug, leading to a significant suppression of protein kinase B, extracellular signal-regulated kinase, and caveolin-1 survival pathways during the detached condition [39]. The metastatic behavior of lung cancer cells becomes enhanced when cancer cells undergo EMT. Gigantol has also been reported to possess the ability to suppress EMT in non-small H460 cells by attenuating the activity of ATP-dependent tyrosine kinase, thereby inhibiting the expression of the major EMT transcription factor, Slug, by both decreasing its transcription and increasing its degradation. The inhibitory effects of gigantol on EMT result in a decrease in the level of migration in H460 cells [40]. Erianin, another promising bibenzyl was isolated from the stems of *Dendrobium chrysotoxum*, a plant used as an antipyretic and analgesic in TCM. The antitumor activity has been investigated in estrogen receptor (ER) positive breast cancer by treating T47D cells with erianin and its effects have been evaluated on multiple cancer-associated pathways [41]. The results showed that erianin significantly decreased the viability of T47D cells at 40, 80 and 160 nM after 24 and 48 hours of treatment. At a concentration of 20 nM after 72 hours of treatment, T47D cells viability was also decreased, demonstrating that the treatment with low dosage over a long time could also inhibit the viability of T47D cells. Interestingly, only a high dose of erianin (80 and 160 nM) could inhibit the proliferation of T47D cells. This study also showed that erianin at low dosages (10 and 20 nM) could suppress migration of T47D cells effectively. In addition, erianin induced apoptosis in T47D cells through reducing Bcl-2 expression and activating caspase signaling and also suppressed the expression of CDKs and caused cell cycle arrest. Meanwhile, erianin did not affect the proliferation of normal breast epithelial cell line MCF10A. Wang et al. (2016) explored the effects of erianin on osteosarcoma (OS) and further elucidated the underlying molecule mechanisms [42]. The inhibitory effects and cytotoxicity of erianin in OS cells was evaluated on various OS cell lines (143B, MG63.2, Saos2, and CCHO). Results demonstrated that erianin inhibits cell proliferation and induces cell cycle G2/M arrest in OS cells by regulating cell cycle-related proteins. Furthermore, erianin induced cell apoptosis by activating both extrinsic (expression of downstream apoptotic protein Caspase-8) and intrinsic (Caspase-9) pathways. Erianin also induced autophagy via ROS/JNK signaling pathway. Dendrofalconerol A (DF-A), a bis(bibenzyl) from *D. falconeri*, demonstrated the anoikis-sensitizing and antimigratory activities and significantly inhibited the growth of H460 lung cancer cells in anchorage-independent conditions [43]. Moreover, DF-A suppresses migrating cancer cells via EMT and integrin proteins. The expression of migration-related integrins, including integrin beta1 and integrin alpha4 was significantly reduced in response to DF-A treatment (0.5-5 µM). Also, DF-A was shown to suppress EMT, as indicated by cadherin switch from N- to E-cadherin and decrease of Snail, Slug and vimentin, revealing thus the potential of DF-A as an anti-metastatic agent [44].

Besides bibenzyls, phenanthrenequinones have also drawn attention from anticancer research. Denbinobin, a 1,4-phenanthrenequinone isolated from the stems of *Dendrobium moniliforme*, was explored for its antitumor mechanisms. This compound inhibited K562 cell viability in a concentration-dependent manner with an IC_50_ value of 1.84 µM, caused G2/M phase accumulation in a time-dependent manner and enhanced tubulin polymerization. Furthermore, denbinobin significantly suppressed the expression of Bcr-Abl and phosphorylation of CrkL, a crucial tyrosinase kinase and an adaptor protein in chronic myeloid leukemia, respectively [45]. Sánchez-Duffhues et al. (2009) investigated antitumor activities of denbinobin and the mechanism involved in Jurkat leukemic cells [46]. This compound inhibits nuclear factor-κB and induces apoptosis via ROS generation, and that this effect takes place in an MAPK-independent pathway. Its cytotoxicity was also examined in several human cancer cells including SK-Hep-1 hepatocarcinoma cells, SNU-484 gastric cancer cells, and HeLa cervix cancer cells with IC_50_ values of 16.4, 7.9 and 22.3 µM, respectively [47]. Moreover, the study revealed that denbinobin inhibits the invasive phenotype of SNU-484 cells suggesting that metalloproteinase (MMP)-2 and MMP-9 may be involved in its anti-invasive activity. A study of Weng et al. (2013) revealed that denbinobin induces human glioblastoma multiforme (GBM) cell apoptosis through IκB kinase inactivation, followed by Akt and forkhead in rhabdomyosarcoma dephos-phorylation and caspase-3 activation signaling cascade. Treatment of GBM cells with denbinobin (0.1-3 µM) reduced cell viability in a concentration-dependent manner. Denbinobin at concentrations of 1 µM and 3 µM significantly decreased the GBM cell viability by 34.1%±3.7% and 62.7%±2.8% (n=3) [48]. Denbinobin, at the concentration of 10 µM, prevents chemokine protein CXCL12-induced prostate cancer (PC-3) cell migration by inhibiting Rac1 activity. The inhibition of Rac1 activity prevented also lamellipodial formation [49]. Moniliformediquinone, another phenanthrenequinone from *D. moniliforme*, have been studied for its tumor inhibition in human hormone refractory metastatic prostate cancer (HRMPC). This compound (3 µM) was found to be a potential anticancer agent for HRMPC by decreasing cellular glutathione, leading to a DNA damage response and cell cycle arrest at the S-phase. Mitochondrial stress also occurs due to moniliformediquinone action through loss of mitochondrial membrane potential and cytochrome c release, which leads to apoptosis [50]. Recently, a bibenzyl-dihydrophenanthrene derivative, named dendrosignatol, together with 3,4-dihydroxy-3,4-dimeth-oxybibenzyl, dendrocandin B, dendrocandin I and dendrofalconerol A, isolated from the whole plant od *Dendrobium signatum*, showed appreciable cytotoxic activity against three human cancer cell lines, including breast cancer MDA-23 1, liver hepatocellular carcinoma HepG2 and colorectal tumor HT-29 cells [51]. Recently, phenanthrene and bibenzyl derivatives were isolated from *Dendrobium nobile*, together with two new spirodiketones, namely (-)- and (+)-denobilone A. All compounds were tested for cytotoxic activities against HeLa, MCF-7 and A549 cells. However, only (-)- and (+)-denobilone A showed moderate inhibitory effects on HeLa, MCF-7, and A459 cells, with IC_50_ values of 9.8, 9.4, and 9.9 µM, respectively. The IC_50_ values of other compounds higher than 10 µM were regarded as inactive [52]. Moderate inhibitory activities against HeLa, MCF-7, and A459 were also assigned to lactone derivatives from *D. nobile*, decumbic acids A and B, (-)-decumbic acid, (-)- and (+)-dendrolactone, together with decumbic acid, 4-(3-hydroxyphenyl)-2-butanone, 3-hydroxy-1(3-methoxy-4-hydroxyphenyl)-propan-1-one and 3’,4’,5’-trimethoxycinnamyl acetate with IC50 values ranging from 15.3 to 30.0 µM [53].

**Figure 1.**
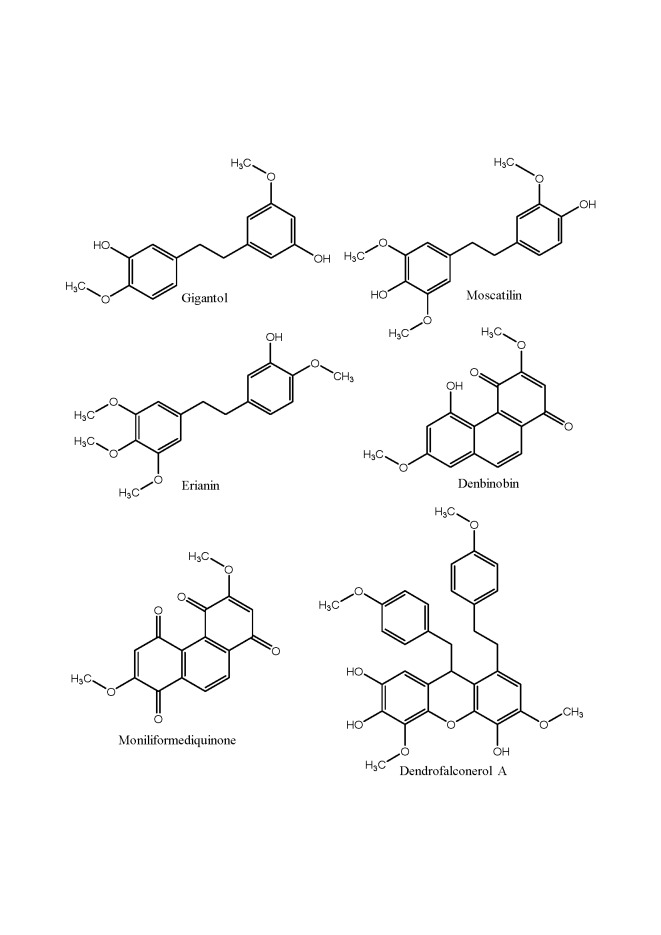
Chemical structures of main anticancer compounds from *Dendrobium* spp.

**Table 1:** *Dendrobium* spp. and their constituents with anticancer properties.

*Dendrobium* species (organ)	Active molecule/type of extract	Biological target	Activity	Refs.
*D. brymerianum* (whole plant)	Methanolic extract Moscatilin Gigantol Lusianthridin Dendroflorin	Human lung cancer cell line H460	Cytotoxic Antimigratory	[31]
*D. candidum* (whole plant)	Methanolic extract Aqueous extract *Not specified*	Human colon carcinoma cell line HCT-116, BALB/c mice bearing 26-M3.1 colon carcinoma cells Azoxymethane- and dextran sulfate sodium-induced colon carcinogenesis in C57BL/6 mice BALB/c mice bearing 26-M3.1 cells Breast cancer cell line MCF-7	Cytotoxic Anti-metastatic Inhibitory Anti-metastatic Anti-proliferative, induction of cell cycle arrest at G2/M phase	[23] [55] [54] [17]
*D. catenatum* (whole plant)	Protein extract Peptides	Human liver cancer cell line HepG2, human gastric cancer cell line SGC-7901 and breast cancer cell line MCF-7	Cytotoxic, anti-proliferative	[26]
*D. chrysotoxum* *(Not specified)*	Erianin	Human mammary gland T47D cells Human osteosarcoma cells 143B and Saos2, BALB/c-nu mice orthotopically inoculated with 143B cells	Antimigratory, anti-proliferative, induction of apoptosis and cell cycle arrest Induction of G2/M-phase arrest, apoptosis and autophagy	[41] [42]
*D. draconis* (stems)	Gigantol	Human lung cancer cell line H460	Antimigratory, induction of apoptosis through mitochondrial-dependent pathway Induction of anoikis, attenuation of epithelial to mesenchymal transition (EMT)	[37, 38] [39, 40]
*D. ellipsophyllum* *(Not specified)*	4,5,4’-trihydroxy-3,3’dimethoxybibenzyl	Human lung epithelial cells H292	Induction of anoikis, attenuation of EMT, antimigratory	[28, 29]
*D. falconeri* (aerial parts)	Dendrofalconerol A	Human lung cancer cell line H460 cells	Antimigratory, induction of anoikis Reduction of the expression of migration-related integrins, suppression of EMT	[43] [44]
*D. fimbriatum* (stems)	Fimbriadimerbibenzyl A, B, E, F, G Moscatilin 4-(3-hydroxy-4-methoxyphenetyl)-2,6-dimethoxylphenol	Human promyelocytic leukemia cells HL-60, human hepatocarcinoma cells SMMC-7721, lung carcinoma cells A-549, colon cancer cells SW480, breast cancer cells MCF-7	Cytotoxic	[30]
*D. formosum* (leaves)	Ethanolic extract	T-cell lymphoma (*in vitro*) Dalton’s lymphoma bearing mice	Cytotoxic, induction of apoptosis Increase of survival time	[24]
*D. loddigesii* *(Not specified)* (stems)	Moscatilin	Human umbilical vein endothelial cells (HUVEC) *in vitro* Human lung adenocarcinomic cells A549 xenograft in nude mice Human breast adenocarcinoma MDA-MB-231 cells *in vitro* and *in vivo* metastatic model HCT-116 *in vitro* and xenograft model *in vivo* Human esophageal cancer cells (squamous and adenocarcinoma)	Anti-proliferative, inhibition of VEGF and bFGF-induced angiogenesis Inhibition of growth of A549 xenograft Inhibitory and antimigratory Induction of apoptosis Growth suppression, induction of apoptosis	[34] [57] [35] [36]
*D. moniloforme* (stems)	Denbinobin Moniliformediquinone	Leukemic cells Human hormone refractory prostate cancer cells PC-3 and DU-145	NF-κB nuclear factor inhibition and apoptosis via ROS generation Glutathione involved mitochondria stress and DNA damage	[46] [50]
*D. nobile* (stems)	Denbinobin (+)- denobilone A (-)-denobilone A decumbic acid decumbic acid A decumbic acid B, (-)-decumbic acid, (-)-dendrolactone, (+)-dendrolactone, 4-(3-hydroxyphenyl)-2-butanone 3-hydroxy-1(3-methoxy-4-hydroxyphenyl)-propan-1-one 3’,4’,5’-trimethoxycinnamyl acetate	Human hepatic adenocarcinoma cells SK-Hep-1, human gastric cell line SNU-484 and HeLa cervical cancer cells Prostatic carcinoma cells PC-3 HeLa, MCF-7 and A549 cells	Cytotoxic Inhibition of invasion of SNU-484 phenotype via MMP-2/-9 expression Antimigratory Cytotoxic	[47] [49] [52, 53]
*D. officinale*	Aqueous extractions	MNNG-induced gastric tumorigenesis in rats	Antioxidative effect, modulation of cytokines related to tumorigenesis, induction of apoptosis, chemoprevention for reducing the risk of gastric cancer	[56]
*D. pulchellum* (stems)	Moscatilin Moscatilin Chrysotobibenzyl Chrysotoxine Crepidatin	Lung cancer cells H23 Lung cancer cells	Inhibition of cell motility and invasion via suppression of ROS Anti-metastatic	[32] [33]
*D. signatum* (whole plant)	Dendrosignatol 3,4-dihydroxy-3,4-dimethoxybibenzyl Dendrocandin B Dendrocandin I Dendrofalconerol A	Human breast cancer cells MDA-231, liver hepatocellular carcinoma HepG2, colorectal adenocarcinoma HT-29 cells	Cytotoxic	[51]
*D. sinense* (whole plant)	3,4,3’-trimethoxy-5,4’-dihydroxybibenzyl Aloifoll 5,3’-dihydroxy-3,4-dimethoxybibenzyl Longicornuol A	Human gastric cancer cells SGC-7901, human hepatoma cells BEL-7402, chronic myelogenous leukemia K562	Cytotoxic	[27]
*D. speciosum* (stems)	Methanolic extract	HepG2 cells	Antigenotoxic	[25]

The majority of reported studies deal with *in vitro* evaluations of potential anticancer effects of crude *Dendrobium* extracts or isolated compounds. Only few of them were deepened *in vivo*. Besides the *in vitro* evaluation of *Dendrobium candidum* methanolic extract by Zhao et al. (2014), its anti-metastatic effect was also assessed in mice with tumors propagated by 26-M3.1 colon carcinoma cells. DCME was the most effective for inhibition of lung metastasis at a concentration of 200 mg/kg.[23]. The same *in vivo* model was used by Li et al. (2014), showing preventive effects of *D. candidum* on the formation of lung metastases. The most marked tumor inhibitory rate of 64.5% has been showed at a dose of 400 mg/kg body weight (b.w.) [54]. Wang et al. (2014) also demonstrated that *D. candidum* is effective in the prevention of chemically-induced colon carcinogenesis in C57BL/6 mice, by increasing the serum SOD level and decreasing the levels of pro-inflammatory cytokines IL-6, IL-12, TNF-α and IFN-γ [55]. After *in vitro* cytotoxicity evaluation of *Dendrobium formosum* leaf ethanolic extract, the results of the *in vivo* antitumor activity on Dalton’s lymphoma were expressed as a ratio of the median survival days of the treated and control group (T/C) of DL bearing mice. With a treatment of 150 mg/kg b.w., an effective T/C value of 172% with a significant increase in life span compared to control was obtained. The T/C value reduced to 144% on treating the mice with 175 mg/kg, which indicates that higher dose of treatment with *D. formosum* ethanolic extract may induce toxicity [24]. The effect of *Dendrobium officinale* water extractions was investigated by oral administration (4.8 and 2.4 g/kg) to the rats of the gastric carcinogenesis model. The study suggests that *D. officinale* could downregulate the expression of malondialdehyde and 8-hydroxy-2-deoxyguanosine and up-regulate the activity of glutathione peroxidase as well as IL-2 during N-methyl-N’-nitro-N-nitrosoguanidine-induced gastric tumorigenesis in rats. *D. officinale* also reduced the level of Activin A, Agrin, IL-1α, intracellular adhesion molecule-1, and tissue inhibitor of matrix metalloproteinase-1 and up-regulated the level of IL-10. The results indicate that *D. officinale* shows antioxidant effects, modulates some cytokines related to tumorigenesis, and induces apoptosis [56]. Only two isolated compounds from *Dendrobium* plants were evaluated *in vivo*. Pai et al. (2013) showed the antimigratory and anti-metastatic effects of moscatilin on human breast cancer in an MDA-MB-231 metastatic model. Moscatilin (100 mg/kg) significantly suppresses breast cancer metastasis to the lungs and reduced the number of metastatic lung nodules and lung weight without causing any toxicity [57]. In the study of Wang et al. (2016), an orthotopic osteosarcoma (OS) model was established by intra-tibial injection of 143B cells to confirm the antitumor effect of erianin *in vivo*. The mice were injected with erianin (2 mg/kg) while the control group was injected with 5% DMSO intraperitoneally every other day for seven times in total. Erianin markedly inhibited growth of OS with no major organ-related toxicity [42].

Given the reported data, phenanthrene derivatives, particularly bibenzyls, seem to be very promising compounds, suggesting anticancer activities (Fig. 1). However, many studies do not investigate more than their cytotoxicity against cancer cell lines *in vitro*, concluding that it suggests their potential for the development of an anticancer drug (Table 1). To the best of our knowledge, no anticancer treatment from *Dendrobium* herbs has been fully developed for instance.

## Neuroprotective effects

Neuroprotection is a vast term involving the preservation of neuronal structure and/or function. There are many central nervous system (CNS) disorders including neurodegenerative diseases, brain ischemia, neurotoxin consumption and traumatic brain injury. Ischemic brain injury and neurodegenerative diseases, such as Alzheimer’s and Parkinson’s diseases, are a problem of growing importance in an aging population. Searching for promising neuroprotective agents aims to prevent or slow disease progression and secondary injuries by slowing the loss of neurons. Despite the fact, that molecular pathways involved in neurodegenerative disorders of CNS are multifaceted and complex, common mechanisms include increased levels in oxidative stress, mitochondrial dysfunction, inflammatory processes or protein aggregation and hyperphosphorylation [16, 58, 59]. Comparing to studies on anticancer effects of *Dendrobium* herbs, there are only a few which have been focused on neuroprotective effects during the last decade. The reported active substances are alkaloids, bibenzyl derivatives and phenolic glucosides. *Dendrobium nobile* alkaloids (DNLA) were studied for their effects on rat primary cultured neurons subjected to oxygen-glucose deprivation/reperfusion (OGD/RP) *in vitro*, in an attempt to attenuate neuronal damage in ischemic brain vascular diseases. Treatment with DNLA (0.025-2.5 mg/mL) significantly attenuated neuronal damage, with evidence of increased cell viability, decreased cell apoptosis, and decreased cell morphologic impairment. DNLA have protective effects against OGD/RP-induced neuronal damages in rat primary neuron cultures by stabilizing mitochondrial membrane potential, inhibiting intracellular free calcium overload, and lessening neuron apoptosis mediated by down-regulating mRNA expression of caspase-3 and caspase-12 [59]. Furthermore, DNLA were studied for their protective effects in neuroinflammation as potential mechanism involved in Alzheimer’s disease (AD) [16]. AD is characterized by the buildup of two aberrant protein aggregates in the brain, i.e. the amyloid plaques composed of amyloid β peptide (Aβ) and neurofibrillary tangles consisted of hyperphosphorylation tau protein [60]. Among a number of pathological abnormalities in AD, inflammatory processes and Aβ deposition trigger neuroinflammatory response, leading to the loss of neurons and the decline of cognitive functions. Thus, Li et al. (2011) reported inhibitory effects of DNLA on memory impairment induced by liposaccharide (LPS) in rats. Indeed, LPS, an inflammation inducer, has been reported to influence Aβ deposition, and LPS injection to the mouse brain ventricle caused memory deficiency and Aβ accumulation. It was clearly demonstrated, that DNLA treatment (40, 80, 160 mg/kg/d for 7 days) significantly protected the rat brain from LPS-induced (50 µg) neuroinflammation and cognitive dysfunction. This effect appears to be due, at least in part, to suppression of LPS-induced overexpression of hippocampus tumor necrosis factor receptor 1 (TNFR1), possibly through inhibition of phosphorylated p38 mitogen activated protein kinases (p-p38 MAPK) and the downstream NF-κB signal pathway [16]. Nie et al. (2016) investigated protective effects of DNLA on Aβ peptide segment 25-35 (Aβ_25-35_)-induced neuron and synaptic loss in mice [61]. 10 µg of Aβ_25-35_ was injected into the bilateral ventricles of male mice followed by an oral administration of DNLA (40 mg/kg) for 19 days. The results showed that DNLA significantly attenuated Aβ_25-35_-induced spatial learning and memory impairments in mice. DNLA prevented Aβ_25-35_-induced neuronal loss in the hippocampus and cortex, increased the number of Nissl bodies, improved the ultrastructural injury of neurons and increased the number of synapses in neurons. Consequently, DNLA can prevent neuronal apoptosis and synaptic loss. The neuroprotective mechanism may also be associated to the inhibition of hyperphosphorylation of tau protein, which could attenuate the dementia symptoms in AD. Yang et al. (2014) investigated the protective effect of alkaloids enriched extract from *D. nobile* (EDNLA) in LPS-induced hyperphosphorylation of tau protein in rat's hippocampus and LPS-induced apoptosis in rat brain. Rats were administrated intragastrically with different doses of EDNLA (20 and 40 mg/kg) every 8 hours for one day, followed by LPS (100 μg), injected to the bilateral ventricle. Two hours after injection with LPS, the phosphorylation of tau protein in hippocampus was observed by western blotting. EDNLA groups (20 and 40 mg/kg) obviously decreased the phosphorylation of the tau protein. Additional rats were treated by EDNLA thrice daily for one week, to examine the effects on LPS-induced apoptosis in the brain. EDNLA treatment decreased the number of apoptotic cells around hippocampus in LPS-treated groups. EDNLA showed beneficial effects against LPS-induced rat AD model and had anti-apoptosis action in rat's brain [58].

**Table 2:** *Dendrobium* spp. and their constituents with neuroprotective activities.

*Dendrobium* species (organ)	Active molecule/type of extract	Biological target	Activity	Refs.
*D. auranticum* var. *denneanum* (stems)	(-)-(7S,8R,7'E)-4-hydroxy-3,3',5,5'-tetramethoxy-8,4'-oxyneolign-7'-ene-7,9,9'-triol 7,9'-bis-*O*-β-D-glucopyranoside (-)-syringaresionl-4,4'-bis-*O*-β-D-glucopyranoside	PC12 cells derived from a pheochromcytoma of the adrenal medulla	Neuroprotective activity against glutamate-induced toxicity	[66]
*D. nobile* *(Not specified)* (stems)	Alkaloids (DNLA)	Primary culture of rat cortical neurons (*in vitro*) Rat’s hippocampus Tau protein in rat's hippocampus Aβ_25-35_-induced spatial learning and memory impairments in mice	Attenuation of neuronal damage on cortical neurons injured by oxygen-glucose deprivation/reperfusion Inhibition of LPS-induced memory impairment Inhibition of hyperphosphorylation and LPS-induced apoptosis Prevention of Aβ_25-35_-induced neuronal and synaptic loss	[59] [16] [58] [61]
*D. crepidatum* (stems)	Crepidatol A Confusarin 3-(2-acetoxy-5-methoxy)-phenylpropanol	PC12 cells	Enhancing activity on NGF-induced neurite outgrowth	[65]
*D. chrysotoxum* (stems)	Chrisotobibenzyl Erianin Chrysotoxine Chrysotoxine	AChE and BChE Bone marrow neuroblastoma cells SH-SY5Y	Enzymatic inhibition Attenuation of 6-OHDA toxicity cells via mitochondria protection and NF-κB modulation Inhibition of the neurotoxicity of 1-methyl-4-phenyl pyridinium (MPP^+^).	[62] [63] [64]

Five bibenzyls and two bibenzyls glucosides were isolated from *Dendrobium chrysotoxum* to investigate their effect on cholinergic neurotransmission in the central and peripheral nervous systems. Acetylcholine and butyrylcholine esterase (AChE and BChE) activity have been used as a marker for cholinergic activity, which plays a crucial role in the learning and memory processes. Indeed, AChE and BChE inhibition may slow neurodegeneration in AD and Parkinson's disease (PD). AChE and BChE inhibitory activities of isolated compounds were assayed using a spectrophotometric method. Chrysotobibenzyl, erianin and chrysotoxine had a certain degree of inhibition ratios against BChE (30.68%, 41.66% and 19.35% of inhibition, respectively) and erianin and chrysotoxine showed weak inhibition ratios against AChE (14.48% and 9.87%, respectively) [62]. The hypothesis that bibenzyls compounds may be neuroprotective against apoptosis induced by the neurotoxins has been tested. Chrysotobibenzyl, crepidatin, chrysotoxine, moscatilin and nobilin B, isolated from *Dendrobium* species, were evaluated for their protective effects against 6-hydroxydopamine (6-OHDA)-induced toxicity in the human neuroblastoma cell line SH-SY5Y. 6-OHDA is a reactive oxidative species (ROS) producing agent which generates hydrogen peroxide, superoxide radical a hydroxyl radical involved in PD. This neurotoxin causes selective death of catecholamine-containing neurons. Chrysotoxine showed a significant effect on the attenuation of 6-OHDA-induced apoptosis in SH-SY5Y cells by regulating ROS initiated multiple signaling pathways [63]. The same way, Song et al. (2012) also demonstrated that chrysotoxine inhibits the neurotoxicity of 1-methyl-4-phenyl pyridinium (MPP^+^). Inhibition of oxidative stress, mitochondrial dysfunction and imbalance in the multiple cell survival/death signaling pathways were involved in its neuroprotection [64].

To show neuroprotective effects of *Dendrobium* isolates, the enhancing activity on nerve growth factor (NGF) induced neurite outgrowth in PC12 cells of ten compounds from *Dendrobium crepidatum* were evaluated *in vitro*. The results indicated that crepiduatol A, confusarin and 3-(2-acetoxy-5-methoxy)-phenylpropanol enhanced the proportion of the NGF-induced (10 ng/mL) neurite-bearing cells at a concentration of 10 μM [65]. A 8,4'-oxyneolignane glucoside and six other phenolic glucosides from *Dendrobium auranticum* var. *denneanum* were assessed for their neuroprotective activity against glutamate-induced toxicity in PC12 cells by an MTT assay. Glutamate induced an inhibition of MTT reduction, while the 8,4'-oxyneolignane glucoside and (-)-syringaresinol-4,4'-bis-*O*-*β*-D-glucopyranoside showed neuroprotective activity at a concentration of 10 μM, with the relative protection of 25.7± 2.2% and 19.3± 5.6%, respectively (the positive control MK-801, 85.9± 3.2%). The results indicated that both compounds may be effective in treating neurodegenerative disorders [66].

To assess neuroprotective effects of active substances from *Dendrobium* herbs, the investigations led during the last decade have been limited to classical molecular targets (Table 2). However, the wealth of molecular information now known about neurodegenerative diseases progression leads to questioning on the efficacy of developed treatments. While Alzheimer targets, for example, the recent discovery that Aβ aggregates at lipid rafts and likely forms neurotoxic pores has led to a new paradigm regarding why past therapeutics may have failed and how to design the next round of compounds for clinical trials [67]. It has been suggested that immunity may contribute to the development of AD and may itself be targeted in future treatments [68]. Therefore, in light of reported immunostimulating activities detailed below, *Dendrobium* plants could be promising therapeutic agents in the next decades.

## Anti-diabetic properties

Diabetes mellitus is a chronically metabolic disorder with abnormally high levels of blood glucose (hyperglycemia), which is caused by the deficiency in insulin secretion and/or the decreased response of the organs to insulin. The worldwide incidence of diabetes type 1 and 2 is dramatically increasing in aging population, becoming a global health issue. Available treatments, such as chemically synthetic agents are expensive with some side effects and toxicity. Thus, the investigations for new treatments have been focused on natural compounds with hypoglycemic or anti-glycation activities, α-glucosidase inhibitors etc.

Some *Dendrobium* species have been investigated in such a direction. Suggesting that *Dendrobium* polysaccharides have the potential to be developed into natural hypoglycemic agents, four polysaccharides from *Dendrobium huoshanense*, *D. officinale*, *D. nobile* and *D. chrysotoxum* were extracted to compare their hypoglycemic activities in alloxan-induced diabetic mice (200 mg/kg b.w.) by oral administration (200, 100 and 50 mg/kg b.w. of *Dendrobium* polysaccharides). The hypoglycemic activities were based on the serum indices including blood glucose, glycosylated proteins and insulin levels, the changes in pathological morphology of the pancreas. The results indicated that the oral administration of polysaccharides from *D. huoshanense, D. officinale* and* D. nobile*, but not *D. chrysotoxum*, presented significant hypoglycemic activities, at all tested dosages, by the evidence of the decreased levels of fasting blood glucose and glycosylated serum protein as well as the increased level of serum insulin in alloxan-induced diabetic mice. The hypoglycemic effect of the three species could be associated with the successful renovation of injured pancreatic islets, which was reflected not only by the histopathological changes of the pancreas but also by the recovery of decreased insulin release. The observed hypoglycemic effects of four *Dendrobium* polysaccharides decreased in the order of *D. huoshanense* > *D. nobile* >* D. officinale* >* D. chrysotoxum* [15]. No hypoglycemic effect was found in alloxan-induced mice that were orally administered with *D. chrysotoxum* in spite of the fact that Zhao et al. (2007) previously reported a significant effect on the reduction in blood glucose level by *D. chrysotoxum* polysaccharide (DCP) in the same mice model [18]. The difference between these two hypoglycemic activities of DCP might result from different lavaging dosages, treatment time and extraction procedure of DCP. Indeed, Zhao et al. used DCP at the concentration of 200-500 mg/kg b.w. on diabetic mice for 7 days, while DCP was orally administered at the concentration of 50-200 mg/kg b.w. for 12 days by Pan et al. [15]. Evidently, it is necessary to use the same animal model, administered with extracts from the same extraction procedure at the same dosages for the same period to correctly evaluate the capability of different *Dendrobium* polysaccharides to decrease blood sugar levels.

Four polyphenols from *Dendrobium loddigesii*, namely loddigesiinols G-J, together with crepiduatol B were evaluated for the α-glucosidase inhibitory activity using spectrophotometric method. These five compounds showed a strong inhibitory activity with IC_50_ values from 2.7 to 18.9 μM, which was significantly stronger than *trans*-resveratrol used as a positive control (IC_50_ of 27.9 μM) [69]. A flavonol glycoside (5-hydroxy-3-methoxy-flavone-7-*O*-(*β*-D-apiosyl-(1-6))-*β*-D-glucoside), as well as gigantol from *Dendrobium devonianum* were also reported as inhibitors of α-glucosidase with the inhibition rate of 43.4% and 36.7%, respectively [70].

Recently, Xu et al. (2013) presented a theoretical basis for therapy of type-2 diabetes by searching for potential drug targets in the genome, using gene chip, also called cDNA microarray investigation, which refers to the many specific gene fragments fixed to a solid support [71]. The gene expression profile, as well as blood sugar lowering and lipid-lowering molecular mechanisms of *Dendrobium* mixtures in diabetic rat model were examined. Three types of Dendrobium mixtures containing *Dendrobium officinale* together with astragalus, *Schisandra*, salvia, arrowroot etc. in different proportions were prepared and administrated to diabetic rats over a total period of 48 days. To realize the microarray test, total RNA was extracted from 1g of liver tissue. Target genes were mainly related to metabolic processes, cell growth, apoptosis, signal transduction, carbohydrate synthesis, carbohydrate degradation, transcription regulation, olfactory receptors, the microtubule system and the nervous system. The cluster of differentiation (CD) gene family and toll-like receptor-9 protein (TLR9) were affected by the treatment of *Dendrobium* mixtures. TLR9 receptors in diabetic rats had low expression but returned to normal levels after treatment. This indicated that *Dendrobium* mixtures could improve autoimmune activity in diabetic rats, thereby enhancing the ability of the body to counter external pathogens and stimuli. This study also found a variety of receptors in the CD gene family with increased levels of expression in the liver of diabetic rats. Overexpression of CD proteins in diabetic patients provides a fertile environment for tumor growth and differentiation. *Dendrobium* mixtures therapy not only changed gene expression patterns in type 2 diabetes but also improved immune activity and reduced the likelihood of cancer development. One of the molecular markers of diabetes is Tspan8 gene. In this study, a three-fold increase of Tspan8 expression was observed in the diabetic rats. Normal expression of Tspan8 was restored after drug treatment, suggesting that this gene could be one of the target sites for *Dendrobium* mixtures treatment.

Non-enzymatic protein glycation is a complicated reaction process between aldehydic group in reducing sugar and amino group in proteins and ultimately gives the advanced glycation end (AGEs) products, which might alter the structures, functions and stability of peptides and are implicated in diabetic complications, such as diabetic cataract and diabetic artherosclerosis, or even skin disorders, such as delayed wound healing [72]. *In vitro* anti-glycation activity was assigned to a water-soluble polysaccharide from *D. huoshanense* (DHP-W2), using a spectrophotometric method, in dose and time dependent manner. DHP-W2 mainly consisted of glucose, xylose, galactose and trace of galacturonic acid, and its average molecular weight was approximately 73 kDa. The inhibition of protein glycation by DHP-W2 reached 23% at the concentration of 0.5 mg/mL after 3 weeks of reaction as against the same reaction time with vitamin C at the concentration of 0.3 mg/mL, which inhibited protein glycation by 28% [73]. Zha et al. (2013) [74] reported that *D. huoshanense* polysaccharide molecular weight decrease, using pectinase hydrolysis, improved its inhibitory action on nonenzymatic glycation of proteins. The sulfation occurred to C-2 and C-6 glucosyl residues seems also to be beneficial to enhance this activity [75]. Diabetic retinopathy (DR) is the most common and serious complication of diabetes. DR leads to the process of vision loss and even blindness, thus the prevention and treatment of DR is a great challenge enhanced by the complexity of this pathogenesis. Angiogenesis plays a major role in the pathogenesis of proliferative DR. Gong et al. (2014) [76] explored the amelioration of ethanol extract of *Dendrobium chrysotoxum* on streptozotocin (STZ)-induced DR and its engaged mechanisms. The findings showed that *D. chrysotoxum* ethanolic extract can alleviate retinal angiogenesis during the process of DR via inhibiting the expression of vascular endothelial growth factor (VEGF) and VEGF receptor 2 in diabetic rats, and some other pro-angiogenic factors such as MMP 2/9, platelet-derived growth factor A/B, basic fibroblast growth factor, insulin-like growth factor. Moreover, *D. chrysotoxum* extract can also ameliorate retinal inflammation via inhibiting NFκB signaling pathway. Decreased retinal mRNA expression of tight junction proteins (including occluding and claudin-1) in diabetic rats was also reversed by *D. chrysotoxum* extract [77]. Concerning *Dendrobium* isolates, the inhibitory mechanism of erianin has been investigated on retinal neoangiogenesis and its contribution to the amelioration of DR. It has been demonstrated, that erianin inhibits retinal neoangiogenesis by abrogating high-glucose-induced VEGF expression by blocking ERK1/2 (a subfamily member of MAPKs)-mediated hypoxia-inducible factor 1-alpha (HIF-1α) activation in retinal endothelial and microglial cells, and further suppressing VEGF-induced activation of VEGFR2 and its downstream signals in retinal endothelial cells [78]. Among other diabetic complications, diabetes-induced cardiomyopathy (DCM) mediated by hyperglycemia can induce the adverse architectural remodeling of heart, and ultimately lead to heart failure and death. Zhang et al. (2016) investigated the effects and the possible mechanisms of the *Dendrobium officinale* extracts (DOE) on diabetic cardiomyopathy in streptozotocin (STZ)-induced mice (at the dose of 50 mg/kg b.w. for 5 consecutive days) [79]. The diabetic mice were received DOE force-feeding once a day for 8 weeks (75, 150 and 300 mg/kg). The results indicated that pretreatment with DOE decreased the heart-to-body weight ratio (HW/BW) and showed an evident hypoglycemic effect. DOE pretreatment significantly decreased creatine kinase, lactate dehydrogenase, total cholesterol and triglyceride levels, limited the production of malonaldialdehyde and increased the activities of superoxide dismutase. The study suggested that DOE possesses the cardioprotective potential against diabetic cardiomyopathy, which may be due to the inhibition of oxidative stress, cardiac lipid accumulation, pro-inflammatory cytokines and cardiac fibrosis. The aim of another *in vivo* study was to determine the preventive effect of *D. officinale* (DO) on the early complications of STZ-induced diabetic rats [80]. DO (1 g/kg/day) was orally administered for 5 weeks, then total cholesterol (TC) and triglyceride (TG), urea nitrogen (BUN), creatinine (CREA) and glutathione peroxidase (GSH-PX) levels were determined, and electroretinographic activity and hypoalgesia were investigated. Treatment with DO significantly attenuated serum levels of TC, TG, BUN and CREA, attenuated the electroretinogram deficits and pathological changes in the eyes of diabetic rats. Moreover, the results indicate that treatment of diabetic rats with DO was effective in improving punctuate mechanical nociception, reduced hypoalgesia and histopathological changes of vital organs induced by hyperglycemia. The protective effect of DO in diabetic rats may be associated with its antioxidant activity, as evidenced by the marked increase in the serum level of GSH-PX. However, DO had no significant effect on blood glucose levels and bodyweight of diabetic rats. In conclusion of this study, DO supplementation is an effective treatment to prevent STZ-induced diabetic complications.

Diabetes mellitus is a chronic disease which a worldwide phenomenon. The cost of diabetes care, both direct and indirect, was $245 billion in 2012. Fifty-nine percent of the direct medical cost was for the population aged 65 and over [81]. Therefore, searching for effective anti-diabetic substances is topical and important in order to prevent morbidity in aging population. *Dendrobium* extracts, polysaccharides and bibenzyls showed promising activities on various anti-diabetic targets (Table 3). However, explored a lead of anti-diabetic ingredients from *Dendrobium* plants would be more extensive to raise a hope of their development as a treatment.

**Table 3:** *Dendrobium* spp. and their constituents with anti-diabetic properties.

*Dendrobium* species (organ)	Active molecule/type of extract	Biological target	Activity	Refs.
*D. chrysotoxum* *(Not specified)* (stems)	Ethanolic extract Erianin Polysaccharide	Diabetic retinopathy Alloxan-induced diabetic mice	Amelioration of retinal angiogenesis Preventing retinal inflammation and tight junction protein decrease Inhibition of high-glucose-induced retinal angiogenesis Inhibition of the increase in blood sugar level	[76] [77] [78] [18]
*D. devonianum* (whole plant)	5-hydroxy-3-methoxy-flavone-7-*O*-(*β*-D-apiosyl-(1-6))-*β*-D-glucoside Gigantol	α-glucosidase *in vitro*	Enzymatic inhibition	[70]
*D. huoshanense* (stems)	Polysaccharides	Alloxan-induced diabetic mice Protein glycation *in vitro*	Hypoglycemic Anti-glycation activity	[15] [73]
*D. loddigesii* (stems)	Loddigessinol G-J Crepidatuol B	α-glucosidase *in vitro*	Enzymatic inhibition	[69]
*D. nobile* (stems)	Polysaccharides	Alloxan-induced diabetic mice	Hypoglycemic	[15]
*D. officinale* (stems)	Polysaccharides Mixture with other TCM herbs Fresh juice Aqueous extracts (after pre-extraction with petroleum ether and 80% ethanol)	Alloxan-induced diabetic mice Genome STZ-induced mice	Hypoglycemic Gene expression of mechanisms involved in type 2 diabetes Cardioprotective potential against diabetic cardiomyopathy STZ-induced diabetic complications, reduction of hypoalgesia and histopathological changes of vital organs induced by hyperglycemia	[15] [71] [79] [80]

## Immunomodulatory properties

Under the effect of aging, immunity becomes slower to respond, autoimmune disorders may develop which can ultimately destroy healthy body tissues [82]. Therefore, action on the immune system in prevention of many age-related diseases becomes an interesting lead explored by more and more scientists. We are facing a new paradigm that some age-related diseases could be prevented by stimulating the immunity. Polysaccharides are the main effective ingredients of *Dendrobium* species with the strongest immunomodulatory activities (Table 4). Several studies report diverse investigations of their activities related to immunostimulation or immunoregulation *in vitro* and *in vivo*.

The effects of crude polysaccharides from five species of *Dendrobium* on macrophage function, such as, promoting phagocytosis, release of NO and cytokines IL-1α, IL-6, IL-10 and TNF-α, were investigated. Polysaccharides from *Dendrobium officinale* exerted the strongest immunomodulatory activities on mouse macrophage line RAW 264.7 cells [83]. A crude polysaccharide fraction (cDOP) has been determined to be the characteristic marker of *D. officinale*. cDOP was destarched and separated into two subfraction polysaccharides, DOPa and DOPb. Both are composed of mannose and glucose at similar ratios and have similar structure with a backbone of 1,4-linked β-D-mannopyranosyl and β-D-glucopyranosyl residues. Significant differences were observed only in their molecular weights. cDOP, DOPa and DOPb were evaluated on RAW254.7 cell line and showed proliferative activities, as well as enhancing TNF-α secretion and phagocytosis in a dose-dependent manner (at the concentrations of 125, 250 and 500 µg/mL). Proliferative effects on lymphocytes alone and with mitogens were also observed. Consequently, DOPa and DOPb were thus proven to be major active polysaccharide markers of *D. officinale* [84]. Xia et al. (2012) also partially characterized polysaccharides from the stem of *D. officinale*; two fractions DOP-1 (533.7 kDa) and DOP-2 (159.5 kDa) were mainly composed of mannose and glucose with a trace amount of galactose and arabinose [85]. The monosaccharide composition of DOP-1 was mannose:glucose:galactose:arabinose 3.13:1.34:0.02: 0.01, and that of DOP-2 was mannose: glucose:galactose:arabinose 3.13:1.24:0.12:0.02. Both, DOP-1 and DOP-2 were evaluated with *in vitro* cell models and the data indicated that they exert significant immunomodulatory effects on innate immune responses mediated by spleen lymphocytes, natural-killer (NK) cells and macrophages. DOP-1 exhibited greater effects on lymphocyte activation while DOP-2 was more effective on macrophage activation. *D. officinale* polysaccharides significantly stimulated the proliferation of splenocytes, DOP-1 was more effective than DOP-2. They also increased NK cell cytotoxicity; it was observed that the optimal polysaccharide concentration was 50 μg/mL, which demonstrates that a high concentration is not essential for strong stimulating effects. He et al. (2016) purified a neutral heteropolysaccharide DOP-1-1 from *D. officinale*, which consisted by mannose and glucose (5.8:1) with an average molecular weight of about 1.78 x 10^5^ Da [86]. A partial structure of DOP-1-1 is an *O*-acetylated glucomannan with β-D-configuration in pyranose sugar forms. Its immunomodulatory activity was evaluated by secretion level of cytokine (IL-1β and IL-10) and TNF-α *in vitro*. The results showed that DOP-1-1 increased significantly the levels of TNF-α and IL-1β in polysaccharide-treated macrophage cells (THP-1) in dose dependent-manner (25, 50 and 100 µg/mL) via the activation of signaling pathways involving ERK1/2 and NF-κB (only at the concentration of 25 µg/mL of DOP-1-1). The aqueous extract (AE) and a purified polysaccharide from the stem of *D. officinale*, Dendronan^®^ (O-acetyl-glucomannan), were examined on RAW 264.7 cells, in order to show whether this is the main component responsible for the immune-stimulating activities. Both, AE and Dendronan^®^ were shown to be non-cytotoxic (even at a high concentration of 160 μg/mL) and can activate macrophages, resulting in enhancing phagocytic activity (Dendronan^®^ at 5-20 μg/mL and AE at 5-40 μg/mL in a dose-dependent manner). Dendronan^®^ at 40 μg/mL and AE at 80 μg/mL stimulated NO production, and they increased amount of cytokines TNF-α, IL-6 and IL-12 in a dose-dependent manner; Dendronan^®^ showed a significant effect on IL-6 and IL-12 production and a plateau was reached at 80-160 μg/mL. RT-PCR showed that the increases in the secretion of NO, and cytokines are due to the increase in their mRNA expressions. Moreover, Western immunoblotting revealed that Dendronan^®^ was able to up-regulate the expression of iNOS protein. The results suggest that Dendronan^®^ is the active component of *D. officinale* [87].

Several studies were also focused on structure and bioactivities of polysaccharides from *Dendrobium huoshanense*. Zha et al. (2007) investigated a polysaccharide fraction HPS-1B23 obtained from a water-soluble polysaccharide HPS from the stems of *D. huoshanense* [88]. HPS-1B23 consists of glucose, mannose and galactose in the ratio of 31:10:8. The *in vitro* study showed that HPS-1B23 possessed an enhancing effect on TNF-α production and the peak of 1130.4 pg/mL was obtained in the culture medium with 200 μg/mL. Two types of immune responses are separately regulated by cytokines that control two general subsets of helper cells known as Th1 and Th2. IFN-γ is evaluated as representative Th1 cytokines mainly secreted from Th1 cells. The highest value of IFN-γ was found in splenocyte culture medium treated with 200 mg/mL of HPS-1B23 in the presence of Concanavalin A (ConA) by the end of culture, which was 3.6-fold that of the blank experiment, suggesting that this polysaccharide has a strong immunostimulating activity. Based on the precedent study, the same research group had an objective to understand the detailed underlying mechanisms of a purified polysaccharide from *D. huoshanense* (DHP). For this purpose, the pattern recognition receptor (PRR) of macrophages RAW264.7 responsible for the recognition of DHP was identified. Results showed that DHP significantly stimulated the secretion of NO, TNF-α and IL-1β in a dose-dependent manner from 10 to 200 µg/mL. It was suggested that the plasma membrane of macrophages contains the pattern recognition receptors responsible for recognizing DHP, which further results in the activation of signaling pathways that is relative to the immunostimulatory actions of DHP. This DHP-binding protein was suggested to be Toll Like Receptor 4 (TLR4), the blocking of which may result in the suppression of DHP-induced macrophages activation. After the recognition of corresponding ligand, TLR4 signaling is initiated by its intracellular Toll/interleukin-1 receptor (TIR) domain, which serves as a scaffold for protein-protein interaction, resulting in a downstream signaling cascade, such as NF-κB, MAPKs and PI3/Akt. In this study, it was found that DHP could activate macrophages through NF-κB, MAPKs and PI3 signaling pathways, which is evidenced by not only the changes in the phosphorylation of NF-κB, p38, ERK, JNK and Akt, but also changes in the levels of NO, TNF-α and IL-1β in cells treated with DHP alone or in combination with the specific inhibitors [89]. Hsieh et al. (2008) determined the structure of the active polysaccharide extracted from the mucilage of *D. huoshanense*, which exhibits specific functions in activating murine splenocytes to produce several cytokines including IFN-α, IL-10, IL-6, and IL-1α, as well as hematopoietic growth factors GM-CSF and G-CSF [90]. A structural modification of stem mucilage polysaccharide by deacetylation of 2-*O*-glucomannan was investigated whether it would affect its stimulatory effect on cytokine production. Treatment of cells with deacetylated stem mucilage was found to maintain cell proliferation, but fail to induce cytokine production. The active polysaccharide fraction of the mucilage was determined to have an average molecular weight of ~10kDa for the ratio of glucoside to mannoside (~1:10) and the degree of acetylation (~35%). A polysaccharide extracted from protocorm-like bodies of *D. huoshanense*, DHP-4A, with molecular weight of 2.32 x 10^5^ Da was shown to stimulate RAW 264.7 macrophage cells to secrete NO, TNF-α, IL-6 and IL-10 via activation of MAPKs (p38, ERK, JNK) and translocation of nuclear NF-κB, indicating that this polysaccharide also possesses good immunoregulatory activity. DHP-4A consists of glucose, arabinose, mannose and rhamnose with a molar ratio of 13.8:3.0:6.1:2.1 [91]. Lin et al. (2014) showed that *D. huoshanense* polysaccharides (DH-PS) induced a Th1, Th2, inflammatory cytokines and chemokines in mice *in vivo* and human *in vitro* [92]. DH-PS expanded mouse splenocytes *in vivo* including CD4^+^ T cells, CD8^+^ T cells, B cells, NK cells, NKT cells, monocytes/macrophages, granulocytes and regulatory T cells. DH-PS induced an anti-inflammatory molecule, IL-1ra, in mouse and human immune cells, especially monocytes. It was observed that IL-1ra level induced by DH-PS was significantly higher than that by F3, a polysaccharide extract isolated from another popular Chinese herbal medicine, *Ganoderma lucidum*. *D. huoshanense* polysaccharides (DHP) also showed immunoregulatory activities in mouse intestine, spleen and liver after oral administration (50-200 mg/kg), as indicated by the production of cytokines IFN-γ and IL-4 [93]. Because IFN-γ is Th1-associated cytokine and IL-4 is Th2-associated cytokine, the different enhancements in the levels of IFN-γ and IL-4 secretion caused by DHP in spleen and liver suggested that DHP exerted immunomodulating responses possibly by changing the balance of Th1/Th2.

Extraction methods of polysaccharides may change their chemical properties and so biological activities. Pan et al. (2015) compared immunomodulatory activities of polysaccharides from *Dendrobium chrysotoxum* (DCP), DCP-H obtained by hot water extraction (HWE) and DCP-E by enzyme-assisted extraction (EAE) [94]. In comparison with DCP-H, DCP-E displayed increased total carbohydrate content as well as the decreased contents of total proteins, while their Mw showed no significant difference. DCP-E was composed of arabinose, glucose, mannose and galactose with molar ratio of 0.8:85.1:12.7:1.4, while DCP-H was composed of arabinose, glucose, mannose, xylose and galactose with the molar ratio of 1.7:57.5:38.8:0.8:1.5. In combination with ConA, both DCP-E and DCP-H could stimulate splenocyte proliferation of BALB/c mice with a dose-dependent relationship. DCP-H at the concentrations of 25 and 50 μg/mL provided slightly higher stimulation rate as compared to DCP-E, but there was no significant difference between them (p>0.05). At 100 μg/mL, DCP-H offered lower stimulation rate, and at 100 μg/mL the stimulation rate of DCP-E was significantly higher (p<0.05) than DCP-H at the same concentration. The results showed a significant difference in immunological activity between DCP-E and DCP-H, which may be related to their physicochemical characteristics resulting from different extraction modes.

Investigations focused on polysaccharides from the stems of *Dendrobium nobile* led to the isolation of a pectic polysaccharide DNP-W5, which was composed of mannose, glucose, galactose, xylose, rhamnose and galacturonic acid in molar ratios of 3.1:8.1:8.2:0.6:4.23.9. *O*-acetyl groups represented approximately 6.9%, but where very important for the expression of the immunological activity of DNP-W5, which displayed remarkable immunoenhancing activities on T- and B-lymphocytes [95]. Bioactive tests *in vitro* also revealed that a rhamnoarabinogalactan DNP-W3 with Mw of 710 kDa (galactose, rhamnose and arabinose 3.1:1.1:1.0) and an acetylated galactomannoglucan DNP-W2 (glucose, mannose and galactose 6.1:2.9:2.0) isolated from the stems of *D. nobile* could stimulate ConA and LPS-induced T and B-lymphocyte proliferation and it was thus suggested that both polysaccharides could be potential immunostimulants [96, 97].

Several* in vivo* studies of immunomodulatory activities of *Dendrobium* polysaccharides were also reported. Different dosages (not precised) of *D. officinale* and its polysaccharides were orally administrated to healthy BALB/c mice. After 4 weeks, delayed-type hypersensitivity and natural killer cell activity (cellular immunity), serum hemolytic complement activity (humoral immunity), macrophage phagocytosis (nonspecific immunity) and interferon-γ production by splenocytes were measured. The results showed that *D. officinale* and its polysaccharides could significantly enhance cellular immunity and nonspecific immunity in mice. Even if humoral immunity was also enhanced by *D. officinale*, the polysaccharides had no influence after oral administration. The production of interferon-γ by murine splenocytes was markedly increased. The molecular weight of the major fraction from the polysaccharides was 533 Da, and was composed of mannose, glucose and rhamnose in a molar ratio of 7.3:1.3:1.0 [98]. Because the immunomodulating effects of *D. officinale* poly-saccharides were individually evaluated using *in vitro* splenic cell or macrophage model after the isolation and purification of single fraction, it is uncertain whether or not they are the most effective fractions in total polysaccharide extracts of *D. officinale*. The aim of the study of Xie et al. (2016) was to identify homogenous polysaccharide fractions with high intestinal immunomodulating activity from *D. officinale* stems [99]. DOP-W3-b was thus obtained through a bioactivity-guided sequential isolation procedure based on *in vitro* Peyer’s patch-mediated immunomodulating activity assay. Peyer’s patches are composed of follicle-associated epithelium containing specialized epithelial cells - M cells and highly specialized lymphoid follicles containing numerous B-cells, T-cells, dendritic cells and macrophages, and have been reported to be essential inductive sites for initiating the intestinal mucosal immune response. Aqueous solutions of DOP-W3-b were orally administered to mice once per day for 3 or 7 days, 500 mg/kg b.w. and 2g/kg b.w. DOP-W3-b time- and dose-dependently stimulated the proliferation of bone marrow cells by the oral administration of 0.5 g/kg or 2g/kg for consecutive seven days. Compared to the control (distilled water administration), the proliferation of bone marrow cells by Peyer’s patch cell supernatant was increased by 19.8% on the third day and 34.2% on the seventh day with 0.5 g/kg/day oral administration, and by 35.2% on the third day and 71.7% on the seventh day with 2 g/kg per day oral administration, suggesting that DOP-W3-b is a potent modulator for the production of hematopoietic growth factor. Structure analysis indicated that DOP-W3-b was composed of mannose and glucose in a molar ratio of 4.5 with a relatively low molecular weight of 1.543x 10^4^ Da. The immunomodulatory activities of Dendronan^®^ *in vivo* in relation to cell mediated and humoral immunity was investigated using a long-term immunosuppressed mice model. The overall results proved that Dendronan^®^ significantly promoted the splenocyte proliferation (at 80 mg/kg BW), corrected the imbalance in spleen lymphocytes subsets ratio (CD3 ratio at 40 mg/kg BW), reversed the diminution of cytokine levels (TNF-α secretion at 80 mg/kg BW), and stimulated the formation of Ig and heamolysin (40 mg/kg BW) [100].

Water-soluble polysaccharide was isolated from the stem of *Dendrobium tosaense* (DTP). Fractioning of DTP produced a neutral polysaccharide fraction DTP-N (87%). DTP and DTP-N consisted of galactose, glucose, and mannose in ratios of 1:9.1:150.7 and 1:12.2:262.5, respectively. DTP-N represents the major portion of DTP, which might explain why the Mw of DTP and DTP-N were similar to each other, with an average Mw of 305 kDa and 221 kDa, respectively. Immunomodulatory effects of DTP administered orally to Balb/c mice for 3 weeks were investigated and it was concluded oral administration of 100 mg/kg and 300 mg/kg of DTP led to a 16.9% and 18.2% increase in the population of splenic NK cells compared with the control group. Moreover, splenocyte proliferation was substantially enhanced by LPS stimulation in a dose-dependent manner in both DTP-treated groups (100 and 300 mg/kg) and the same DTP treatments significantly up regulated the proliferation of splenocytes through ConA stimulation, compared with the control group. However, DTP exerted no effects on the population of T cells, B cells, cytotoxic T cells, or helper T cells [101].

**Table 4:** *Dendrobium* spp. and their constituents with immunomodulatory activities

*Dendrobium* species (organ)	Active molecule/type of extract	Biological target	Activity	Refs.
*D. aphyllum* (stems)	Moscatin Moscatilin Tricetin 3',4',5'-trimethyl ether 7-*O*-β-glucopyranoside	LPS-stimulated RAW 264.7 cells	Inhibition of NO production	[109]
*D. candidum* *(Not specified)*	Water liquid extract	Labial glands from patients with Sjögren syndrome	Increasing of AQP-5, promotion of secretion of saliva to improve dry mouth symptoms	[108]
*D. chrysotoxum* (stems)	Polysaccharides DCP-H and DCP-E	Spleen cells from BALB/c mice	Stimulation of splenocyte proliferation	[94]
*D. huoshanense* *(Not specified)* (stems) (Leaves and stems) (protocorm-like bodies)	Polysaccharides Polysaccharide HPS-1B23 Polysaccharide DHP Mucilage polysaccharide Polysaccharide DHP-4A	Mouse intestine, spleen and liver (*in vivo*) Spleen cells and peritoneal macrophages from BALB/c mice Murine splenocytes RAW264.7 macrophages and peritoneal macrophages C3H/HeN and C3H/HeJ Mouse splenocytes and human peripheral blood mononuclear cells RAW 264.7 macrophage cells	Enhancements in the levels of IFN-γ and IL-4 secretion in spleen and liver by changing the balance of Th1/Th2 Enhancing effect on TNF-α production and IFN-γ secretion Activation of macrophages to release NO, TNF-α and IL-1β, binding of DHP to the surface of macrophages via TLR4 receptor to activate cells via NF-κB, MAPKs and PI3K/Akt signaling pathways Increasing production of IFN-γ, IL-6, IL-10 and IL-1α, induction of hematopoietic growth factors GM-CSF and G-CSF Inducing a panel of cytokines/chemokines in mice *in vivo* and human *in vitro* Stimulation of NO, TNF-α, IL-6 and IL-10 secretion via activation of MAPKs (p38, ERK, JNK) and translocation of nuclear NF-κB	[93] [88] [89] [90] [92] [91]
*D. officinale* *(Not specified)* (stems) (stems) (stems) (stems) (stems) (stems)	Polysaccharides Polysaccharide fractions DOP-1 and DOP-2 Heteropolysaccharide DOP-1-1 Polysaccharide fraction cDOP and its sufractions DOPa and DOPb Polysaccharide fraction DOP-W3-b Dendronan^®^ Polysaccharide DP	Macrophages function Murine splenocytes of BALB/c mice Macrophages function THP-1 cells RAW 264.7 macrophages Mouse spleen lymphocytes Bone marrow cells stimulated by the suspension of Peyer’s patch cells of female ICR mice RAW 264.7 macrophages Sjögren's syndrome model mice A253 cell line	Promoting release of NO, phagocytosis, and cytokines IL-1α, IL-6, IL-10 and TNF-α Enhancing of cellular and nonspecific immunity, increasing IFN-γ production Effects on splenocyte proliferation and NK cytotoxicityn activation of macrophage function Stimulation of cytokine production TNF-α and IL-1β, induction of immune activities via ERK1/2 and NF-κB Enhancing cell proliferation, TNF-a secretion, and phagocytosis Induction of the proliferation Regulation of intestinal mucosal immune activity by changing intestinal mucosal structures, promoting the secretions of cytokines from Peyer’s patches and mesenteric lymph nodes, increasing the production of secretory immunoglobulin A in the lamia propria Enhancing phagocytosis activity, up-regulating NO and influencing cytokine (TNF-α, IL-6 and IL-12) production *In vivo* promotion of splenocyte proliferation, correction of the imbalance in spleen lymphocyte subsets ratio, reversion of the diminution of cytokine levels, stimulation of the formation of Ig and haemolysin Amelioration of the abnormalities of AQP-5 in saliva secretion Inhibition of TNF-α-induced apoptosis	[83] [98] [85] [86] [84] [99] [87] [100] [106] [107]
*D. nobile* (stems)	Polysaccharide DNP-W5 Rhamnoarabinogalactan DNP-W3 and acetylated galactomannoglucan DNP-W2 Polysaccharide DNP4-2	T- and B-lymphocytes S180 mice	Enhancing activities Stimulation of ConA and LPS-induced proliferation Increasing of immune index and promotion of cytokine secretion *in vivo*	[95] [96, 97] [102]
*D. tosaense* (stems)	Polysaccharide DTP	Splenic NK cells, splenocytes in BALB/c mice	Stimulation of the population of splenic NK cells, cytotoxicity, macrophage phagocytosis, and cytokine induction	[101]

Four polysaccharide fractions from *D. nobile*, DNP1-1, DNP2-1, DNP3-1, and DNP4-2, were evaluated for their immunomodulation effects. Mice were injected intraperitoneally once a day over 10 days with 5, 2.5 and 1.25 mg/mL of each polysaccharide. To evaluate the effect of the polysaccharide fractions on the immune system, the spleen and thymus index (weight of spleen/thymus (mg)/body weight (g)) were calculated. All the polysaccharide-treatment groups except DNP4-2 exhibited a lower value of spleen index, which indicates that there was no significant increasing effect of the three polysaccharides on the spleen index in the mice. At the dose of 2.5 mg/mL of DNP4-2, significant increasing was observed, but not in a concentration-dependent manner. All polysaccharide fractions exhibited strong increasing effects on the thymus index, especially for DNP4-2 at the concentration of 2.5 mg/mL, but not in a concentration-dependent manner. Moreover, at this dose, DNP4-2 strongly promoted the secretion of cytokines TNF-α, IFN-γ and IL-2 [102].

Immunomodulatory effects of orally administrated crude polysaccharide fraction of *D. huoshanense* was clinically evaluated on patients with moderate to severe recalcitrant atopic dermatitis (AD). The cause of AD is unknown but believed to involve among others immune system dysfunction. The condition typically starts in childhood with changing severity over the years [103]. Twenty-seven patients aged from 4-18 years that had not responded to topical therapy were treated with polysaccharides from *D. huoshanense* for 4 weeks and followed-up for another 4 weeks. Serum levels of IL-5, IL-13, IFN-γ, and TGF-β1 decreased significantly between weeks 0 and 4 and between weeks 0 and 8. No significant difference in the levels of IL-10 was found. The polysaccharide from *D. huoshanense* reduced the levels of some cytokines associated with atopic dermatitis and had beneficial effects on symptoms. No serious adverse effects occurred when it was administered orally for 4 weeks [104].

Sjögren's syndrome (SS) is a chronic autoimmune disease with exocrine glands disorder with associated lymphocytes infiltration, and is usually clinically manifested as dry eyes and dry mouth [105]. In SS patients, an over-expression of TNF-α, IL-1β and IL-6 is observed in response to immune-mediated inflammation, as well as an abnormality of aquaporin 5 (AQP-5) in saliva secretion. *Dendrobium officinale* polysaccharide (DP) and its possible protective effect on the submandibular gland from the progressive destruction in the experimental SS model mice was examined by Lin et al. (2011) [106]. DP was found to consist of mannose, glucose, galactose, arabinose, xylose and glucuronic acid in the molar ratio of 10:0.25:1.2:4.7:1.3:1.4. Mice were administrated with 200 μL of DP at the dose of 20 mg/mL in physiological saline per day for 25 days. The pro-inflammatory cytokines TNF-α, IL-6, IL-1β and its activator MMP-9, were found over-expressed in the model mice, but significantly suppressed by the treatment of DP. Given that the AQP-5 is suppressed by the TNF-α in the murine lung epithelial cells, it was speculated that the decrease of AQP-5 in the model mice is, at least partially, due to the up-regulated expression of TNF-α. The inhibition of AQP-5 was directly assessed by the addition of human TNF-α on A253 human salivary cell line. Significantly decreased AQP-5 protein levels were observed after treatment with TNF-α 100 units/mL for 8hr, but was reversed by the pre-treatment of DP in a dose-dependent manner. However, even though the DP administration ameliorated the expression of AQP-5 in SG, there was still a fair decrease in the DP group compared with the control group. The results indicated that there might be other factors responsible for the decrease of AQP-5. Furthermore, the protective effect of DP on A253 cell line against TNF-α-induced apoptosis was investigated by Xing et al. 2013 [107]. A253 cells were pre-treated with DP for 12hr before TNF-α addition, then NF-κB, phosphorylation of MAPKs, ROS generation, mitochondrial membrane potential and proapoptotic proteins were examined. In summary, prolonged MAPK activation and translocation of p65 subunit of NF-κB in the nuclei were observed, as well as decreased mitochondrial membrane potential and accumulated cellular ROS, leading to the dramatic up-regulation of apoptotic proteins. Pre-treatment with DP was further demonstrated to be protective in a dose-dependent manner in response to the TNF-α stimulus. In research for potential therapies, sixteen patients with SS suffered from deficient secretion of saliva due to an autoimmune destruction of salivary glands leading to dry mouth symptoms, were orally administered with *Dendrobium candidum* extracted liquid (DCEL) for 1 week. Saliva and salivary glands biopsies from labial glands of patients were collected and examined by immunoreactivity and immunohistochemistry techniques. Results showed that salivary secretion increased by about 65% in patients treated with DCEL as compared with the control group. It was demonstrated that *D. candidum* extract would regulate the expression of AQP-5 in labial glands of SS patients and thereby promoted secretion of saliva to improve dry mouth symptoms [108].

Besides polysaccharides, phenolic compounds from *Dendrobium aphyllum*, namely moscatin, moscatilin and tricetin 3',4',5'-trimethyl ether 7-*O*-β-glucopyranoside, inhibited NO production at the concentration of 25 μM in LPS-stimulated RAW 264.7 cells with the inhibition of 32.48%, 35.68% and 38.50%, respectively [109].

**Figure 2.**
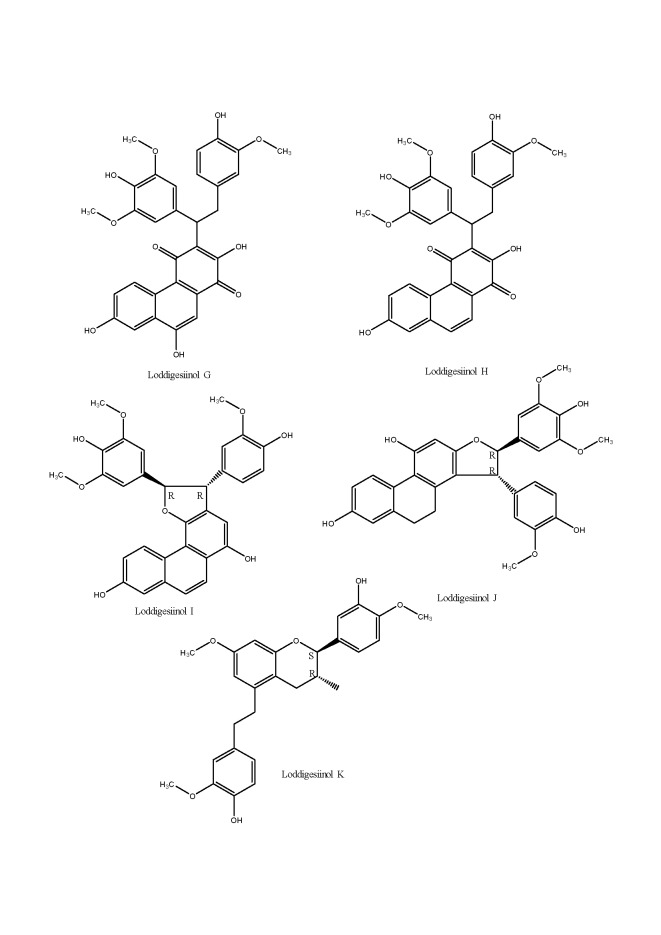
Chemical structures of anti-diabetic compounds of loddigesiinols G-K from *D. loddigesi*.

## Patents

In light of above mentioned scientific papers focused on *Dendrobium* extracts and isolates and their activities associated to aging-related diseases, the existence of patents seems obvious. Since 2005, more than 320 patents have been registered, mostly Chinese, dealing with preparations containing *Dendrobium* plants for conditioning, regulating or alleviating cancer, diabetes or improve human immunity. However, if we look at these inventions closely, we become aware that the majority of them focus on Traditional Chinese medicine prescriptions associated to at least about ten other Chinese herbs, such as *Ganoderma lucidum*, ginseng, licorice, astragalus, *Millettia speciosa* etc., to produce synergism and enhance targeted health benefits (patents CN105441280, CN103566350, CN105748996, CN105494776). The inventions describe various beverages (teas, wines), food, powders, gels, capsules and production methods thereof. The prescriptions are mostly preventive or assisting diabetes treatments, reducing blood glucose, improving yin-deficiency, and accompanying patients with chemotherapy or radiotherapy. Furthermore, the majority of the patented preparations mention only *Dendrobium*, dendrobe or herba dendrobii without precision of the species; some of them contain other orchids included in Chinese Pharmacopoeia, such as *Gastrodia elata* or *Bletilla striata* (patents CN105902844, CN105597027). If we focus on specific *Dendrobium* species, *D. officinale* takes part of an instant powder or healthy liquor enhancing immunity and improving the anti-cancer effect of this species (patents CN105533744, CN105602812). *D. officinale* root extract is also exploited in for controlling high blood sugar and improving sugar tolerance (patent CN105434886). *Dendrobium candidum* preparation with walnut kernels, asparagus roots, *Codonopsis pilosula*, Chinese angelica, *Ligusticum wallichii* and licorice has a synergic effect and can significantly improve body discomfort caused by tumor pain and adverse effects arising from antitumour treatment, particularly lung cancer patients (patent CN104606510). A traditional Chinese medicine containing 29 ingredients of which *Dendrobium nobile*, has been registered to effectively treat diabetes (patent CN105596776). Only few patents deal with *Dendrobium* phytochemicals. Dendrophenol (syn. moscatilin) has been patented as an agent inhibiting cervical cancer cell proliferation and inducing autophagic apoptosis *in vitro* in an effective concentration in the range of 25-200 mol/L (patent CN105943523). Loddigesiinols G-K (Fig. 2) have been registered to be used as hypoglycemic drug for preventing and curing cardiovascular disease (patent CN103664568).

## Conclusion

In this review, we reported the main research on *Dendrobium* species and their most active constituents. Searching for anticancer properties of *Dendrobium* plants, various targets and mechanisms have been explored. Whereas anticancer mechanisms of gigantol, moscatilin, denbinobin or erianin have been explored, there are still many studies, which are satisfied with the evaluation of cytotoxicity against cancer cell lines and miss in depth explorations. To the best of our knowledge, no *Dendrobium* anticancer drug has been subjected to clinical trials. Several species of *Dendrobium* exerted neuroprotective activities, but their evaluation on modern targets should be undertaken in the future. Their immunomodulatory activities are promising and interesting clinical studies on patients with Sjögren syndrome or atopic dermatitis have been reported [104, 108]. In light of recent discoveries about the connections between immunity and diseases developed in the elderly, this lead would continue to be explored and more clinical studies would be realized to confirm *in vitro* and *in vivo* investigations. *Dendrobium* species also showed interesting hits on anti-diabetic targets, but they seem to be more useful against diabetic complications, such as diabetic retinopathy. The existence of several patents has also been mentioned in this review, which is an unusual, but pertinent issue. Even if the majority of the patents are related to mixtures of TCM herbs, including *Dendrobium*, a will of a potential development of acquired knowledge has to be noticed. Whatever happens, given the investigations of *Dendrobium* herbs and their constituents, which have been led during the last decade, an untapped potential for the development of new therapeutic agents for treating age-related pathologies is undeniable.
